# Supplementary material for: First Principles Investigation of C, Cl2 and CO Co-Adsorption on ZrSiO4 Surfaces for Carbochlorination Reaction
Source: Materials (Basel). 2024 Mar 26;17(7):1500. doi: 10.3390/ma17071500 (PMC11012826; doi:10.3390/ma17071500)
Supplement: Supplementary file 1 [file materials-17-01500-s001.zip › materials-2873091-supplementary.pdf]

Support information figure S1:

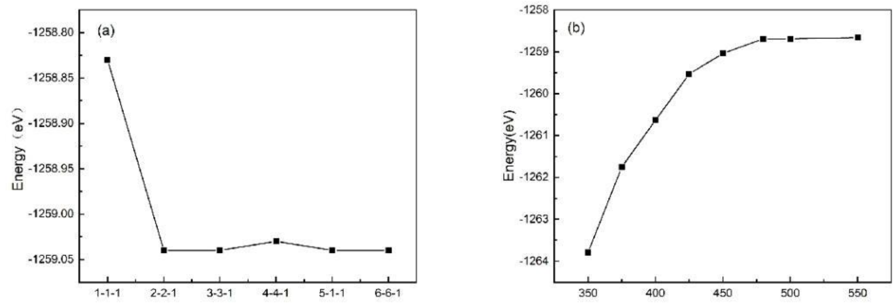


Figure S1:Test results of K-point convergence(a) and truncation energy(b) of the (100) surface of $ZrSiO_4$

Support information figure S2:

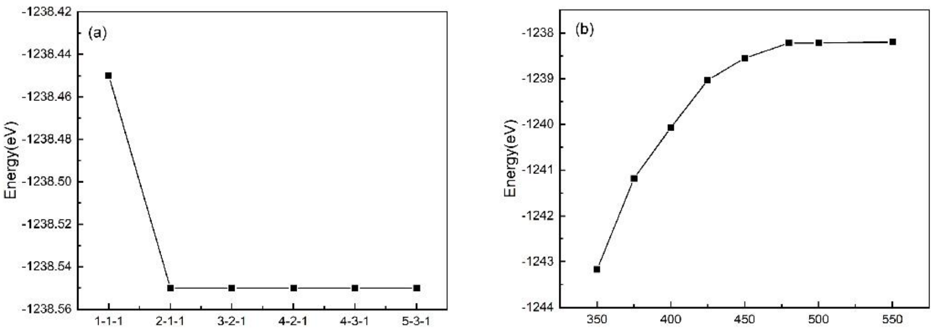


Figure S2:Test results of K-point convergence(a) and truncation energy(b) of the (110) surface of $ZrSiO_4$

Support information figure S3:

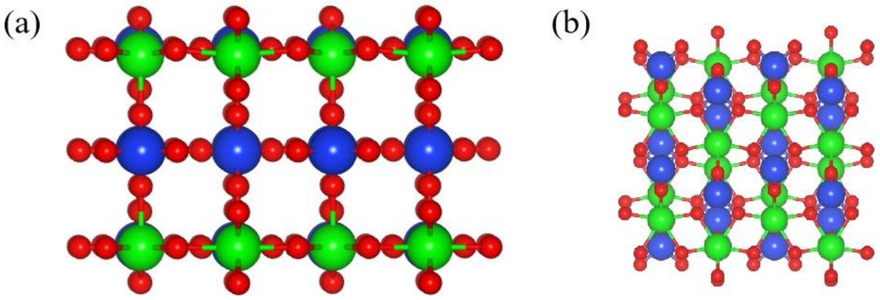


Figure S3: Optimized model structure of the (100) surface of $ZrSiO_4$
: front view (a) and top view (b), where the green ball represents Zr atoms, the dark blue ball represents Si atoms, and the red ball represents O atoms (the same below).

Support information figure S4:

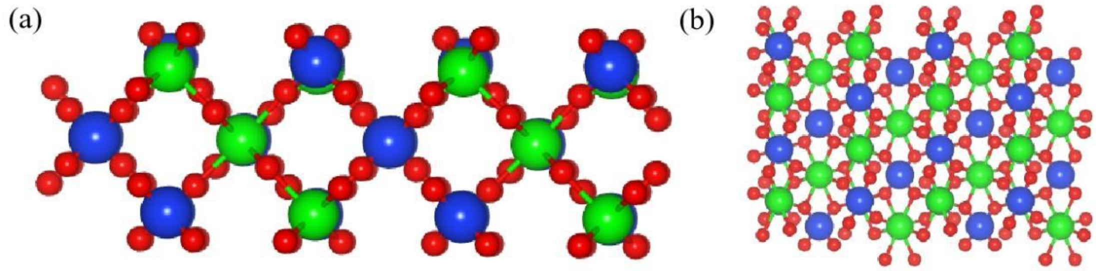


Figure S4: Optimized model structure of the (110) surface of $ZrSiO_4$ : front view (a) and top view (b)

Support information figure S5:

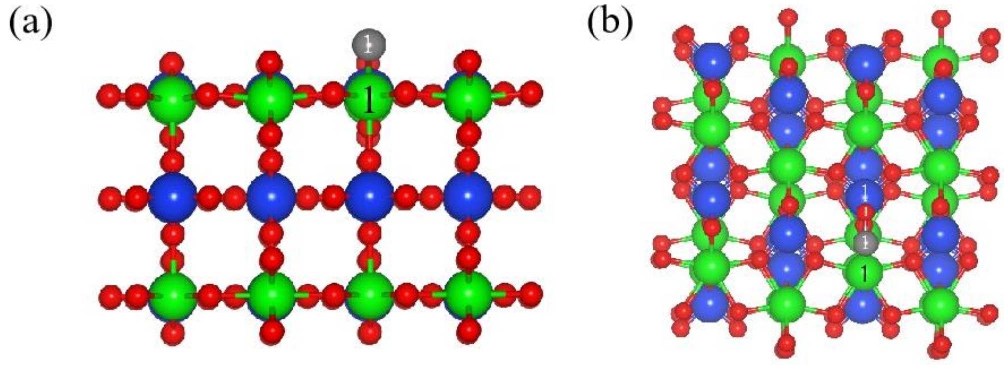


Figure S5: Optimized model structure for the adsorption of C on the (100) plane of $ZrSiO_4$ : front view (a) and top view (b) where the gray balls represent C atoms (the same below)

Support information figure S6:

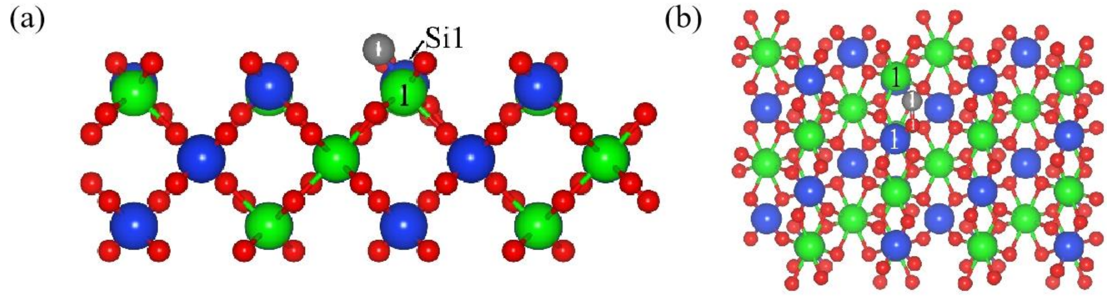


Figure S6: Optimized model structure of C adsorption on (110) plane of $ZrSiO_4$: front view (a) and top view (b)

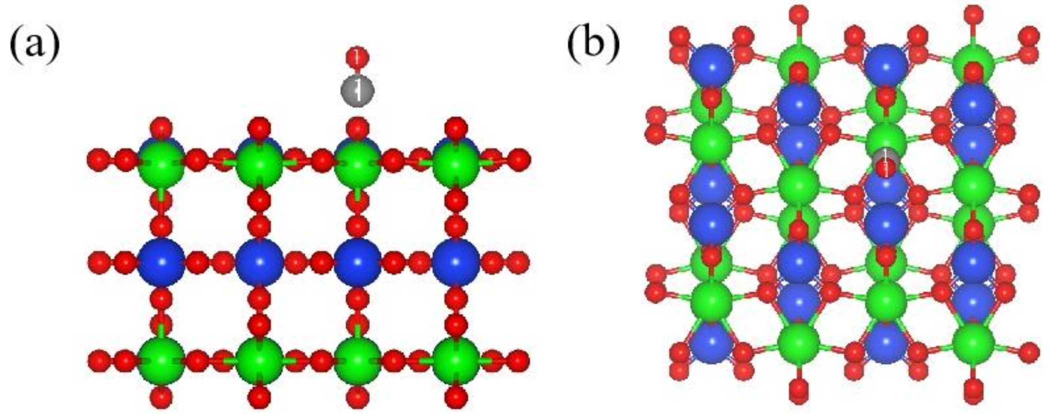


Figure S7: Optimized model structure for the adsorption of CO on (100) plane of $ZrSiO_4$: front view (a) and top view (b)

Support information figure S8:

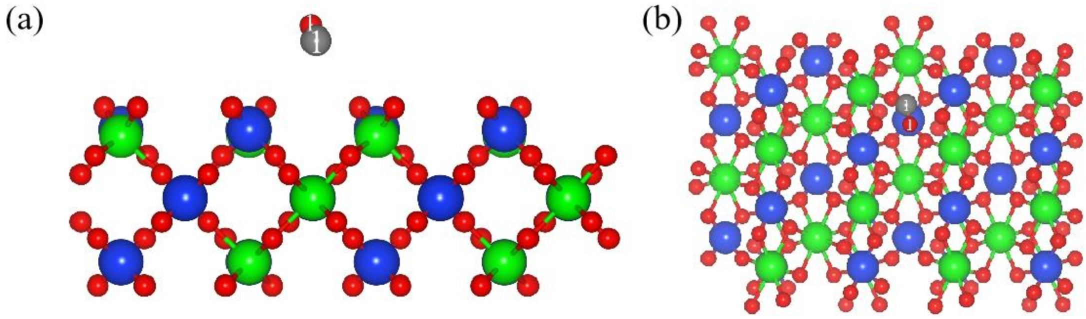


Figure S8: Optimized model structure of CO adsorption on (110) plane of $ZrSiO_4$: front view (a) and top view (b)

Support information figure S9:

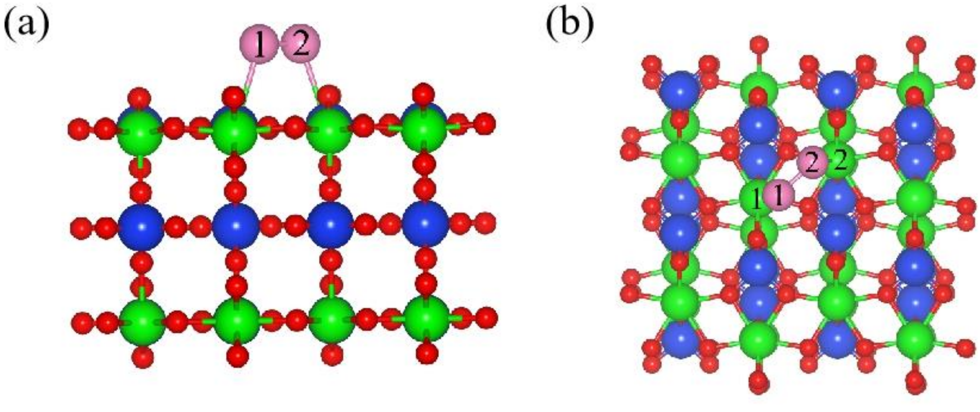


Figure S9: Optimized model structure for the adsorption of $Cl_2$ on (100) plane of

ZrSiO4: front view (a) and top view (b) where pink balls represents Cl atoms (the same below)

Support information figure S10:

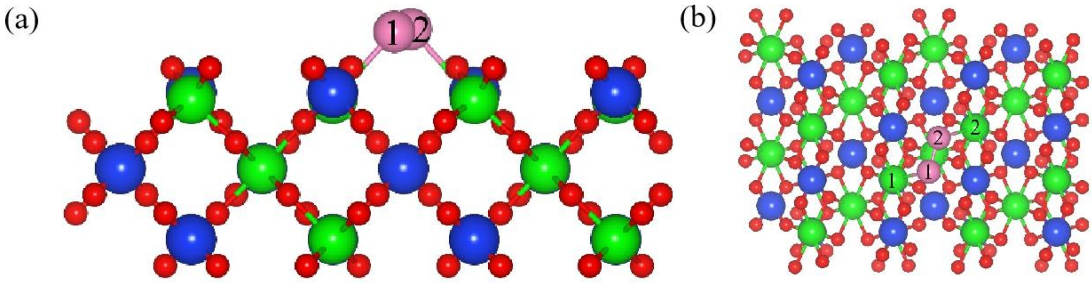


Figure S10: Optimized model structure of $Cl_2$ adsorption on (110) plane of $ZrSiO_4$: front view (a) and top view (b)

Support information figure S11:

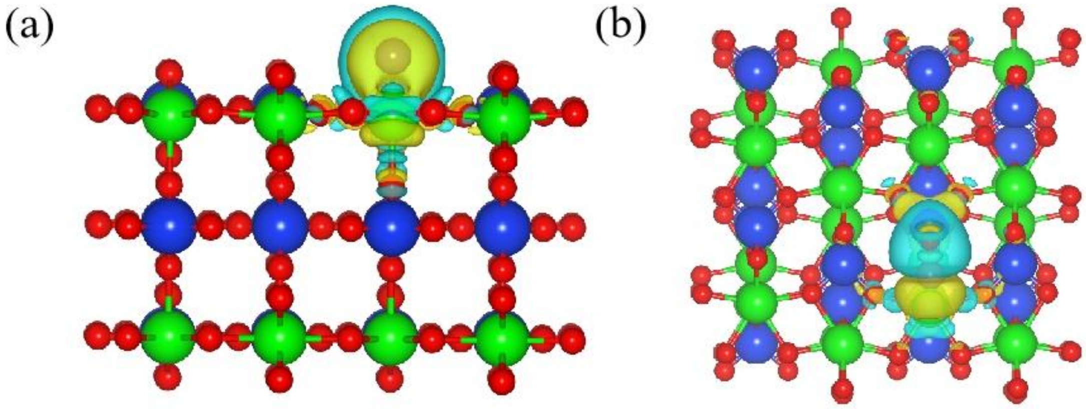


Figure S11: Differential charge density diagram of C adsorption on (100) plane of ZrSiO4 : front view (a) and top view (b),where the blue represent the charge accumulation areas, and the yellow represent the charge depleted areas (the same below)

Support information figure S12:

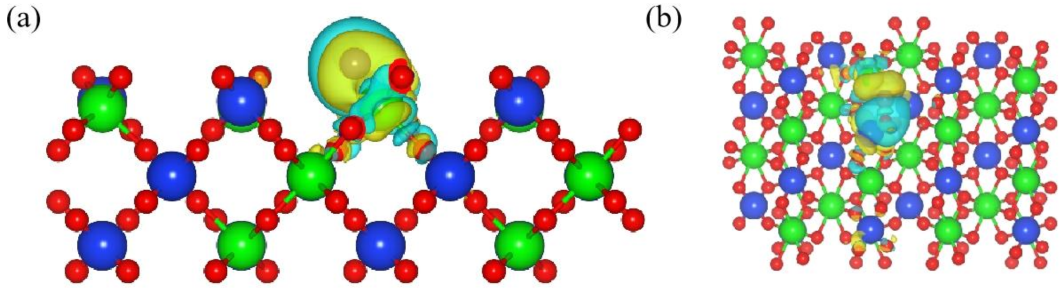


Figure S12: Differential charge density diagram of C adsorption on (110) plane of $ZrSiO_4$: front view (a) and top view (b)

Support information figure S13:

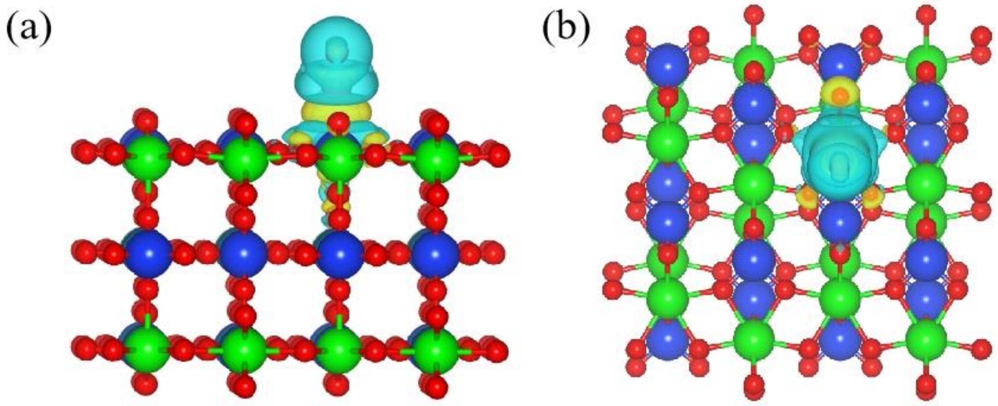


Figure S13: Differential charge density diagram of CO adsorption on (100) plane of $ZrSiO_4$: front view (a) and top view (b)

Support information figure S14:

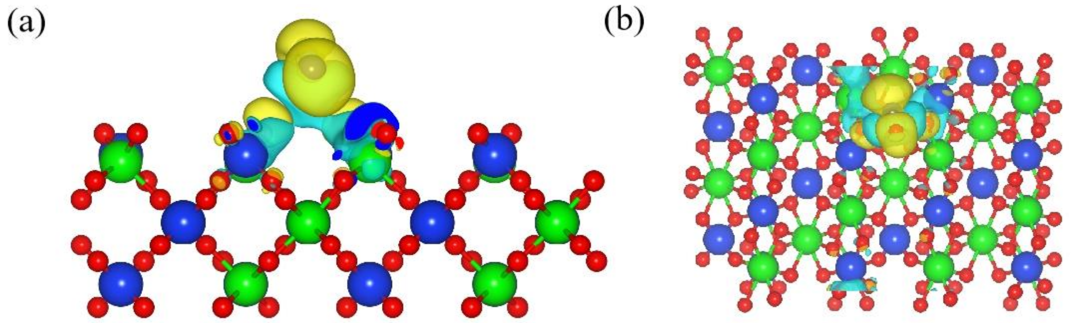


Figure S14: Differential charge density diagram of CO adsorption on (110) plane of $ZrSiO_4$: front view (a) and top view (b)

Support information figure 15:

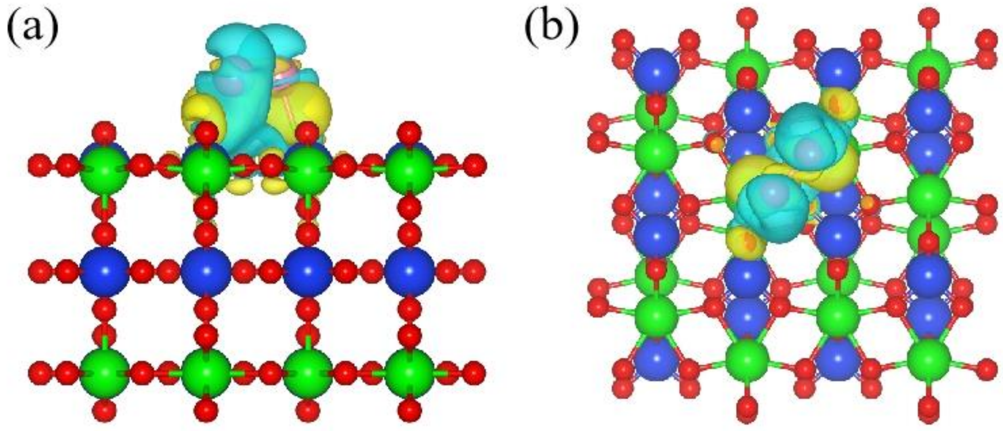


Figure S15: Differential charge density diagram of $Cl_2$ adsorption on (100) plane of $ZrSiO_4$: front view (a) and top view (b)

Support information figure S16:

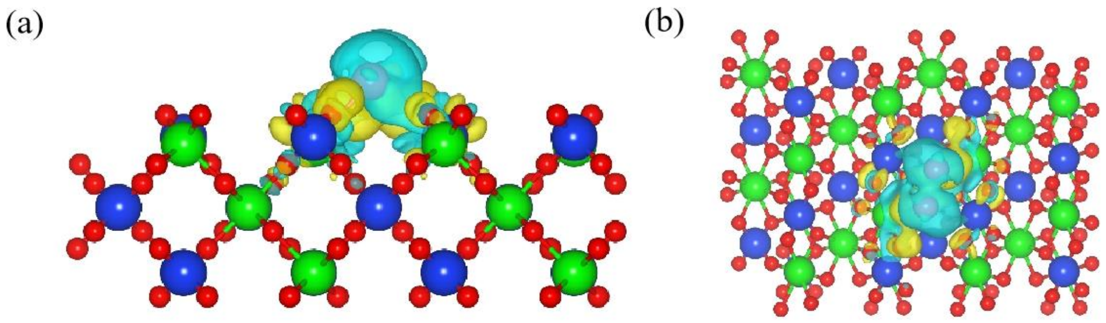


Figure S16: Differential charge density diagram of $Cl_2$ adsorption on (110) plane of $ZrSiO_4$: front view (a) and top view (b)

Support information table S1:

Table S1: Bader charge of C, CO, and $Cl_2$ adsorption on (100) and (110) plane of $ZrSiO_4$

| Adsorption structure | Element | Bader charge |
|---|---|---|
| 100-C | C | -0.17 |
| 100-$Cl_2$ | Cl1 | -0.02 |
| | Cl2 | 0.00 |
| 100-CO | O | 1.05 |
| | C | -1.08 |
| 110-C | C | -0.19 |
| 110-Cl2 | Cl1 | -0.01 |
| | Cl2 | -0.01 |
| 110-CO | O | 1.40 |
| | C | -0.19 |

Support information table S2:

Table S2: Distances between C, CO, and $Cl_2$ and the surface of zirconium silicate (Å) before adsorption

| Adsorption structure | Cl1-surface | Cl2-surface | CO/O-surface | Cl3-surface | Cl4-surface |
|---|---|---|---|---|---|
| 100-C-$Cl_2$ | 2.59 | 2.44 | 1.21 | | |
| 100-CO-$Cl_2$ | 3.01 | 2.46 | 1.72 | | |
| 110-C-$Cl_2$ | 2.48 | 2.49 | 1.90 | | |
| 100-CO-$Cl_2$ | 2.48 | 2.44 | 1.90 | | |
| 110-C-2$Cl_2$ | 2.46 | 2.44 | 1.19 | 2.86(Cl3 and Cl4 form a bond with a bond length of 2.08) | |
| 110-2C-2$Cl_2$ | 2.47 | 2.50 | 1.29 | 2.47 | 1.72 |
